# The Relationship between Procyanidin Structure and Their Protective Effect in a Parkinson’s Disease Model

**DOI:** 10.3390/molecules27155007

**Published:** 2022-08-06

**Authors:** Juan Chen, Yixuan Chen, Yangfan Zheng, Jiawen Zhao, Huilin Yu, Jiajin Zhu

**Affiliations:** Department of Food Science and Nutrition, Zhejiang University, Hangzhou 310000, China

**Keywords:** procyanidins, Parkinson’s disease, PC12 cells, zebrafish, Nrf2/ARE pathway

## Abstract

This study evaluated the effect of grape seed-derived monomer, dimeric, and trimeric procyanidins on rat pheochromocytoma cell line (PC12) cells and in a zebrafish Parkinson’s disease (PD) model. PC12 cells were cultured with grape seed-derived procyanidins or deprenyl for 24 h and then exposed to 1.5 mm 1-methyl-4-phenylpyridinium (MPP^+^) for 24 h. Zebrafish larvae (AB strain) 3 days post-fertilization were incubated with deprenyl or grape seed-derived procyanidins in 400 µM 1-methyl-4-phenyl-1,2,3,6-tetrahydropyridine (MPTP) for 4 days. The results showed that the procyanidin dimers procyanidin B1 (B1), procyanidin B2 (B2), procyanidin B3 (B3), procyanidin B4 (B4), procyanidin B1-3-*O*-gallate (B1-G), procyanidin B2-3-*O*-gallate (B2-G), and the procyanidin trimer procyanidin C1 (C1) had a protective effect on PC12 cells, decreasing the damaged dopaminergic neurons and motor impairment in zebrafish. In PC12 cells and the zebrafish PD model, procyanidin (B1, B2, B3, B4, B1-G, B2-G, C1) treatment decreased the content of reactive oxygen species (ROS) and malondialdehyde (MDA), increased the activity of antioxidant enzymes glutathione peroxidase (GSH-Px), catalase (CAT), and superoxide dismutase (SOD), and upregulated the expression of nuclear factor-erythroid 2-related factor (Nrf2), NAD(P)H: quinone oxidoreductase 1 (NQO1), and heme oxygenase-1 (HO-1). These results suggest that in PC12 cells and the zebrafish PD model, the neuroprotective effects of the procyanidins were positively correlated with their degree of polymerization.

## 1. Introduction

Parkinson’s disease (PD) is the second most prevalent neurodegenerative disease after Alzheimer’s disease [1]. The main pathological signs include degeneration of dopaminergic neurons in the dense part of the substantia nigra, decrease in dopamine content in the striatum, and cellular aggregation of Lewy bodies [2]. Approximately 5 million people suffer from PD every year. The incidence rate in males is higher than in females, and the average age of onset is 60 years old. The prevalence and incidence rates increase exponentially with age [3]. The main symptoms of PD regarding motion include static tremor, myotonia, motor retardation, and postural and gait disorders [4]. In terms of non-motor function, the main symptoms of PD include olfactory disorders, cognitive abnormalities, and sleep and emotional disorders [4,5]. In the substantia nigra of patients with PD, it was found that deoxyribonucleic acid (DNA) and protein were highly oxidized, the level of lipid peroxidation was high, and glutathione was reduced [6,7]. It was also observed that the level of 8-hydroxy-2′-deoxyguanosine, a DNA oxidative marker, was increased [8]. Oxidative stress plays a key role in the loss of dopaminergic neurons [9,10]. Therefore, supplementing antioxidants and eliminating excessive reactive oxygen species (ROS) may be an effective method for preventing PD.

**Figure 1:**
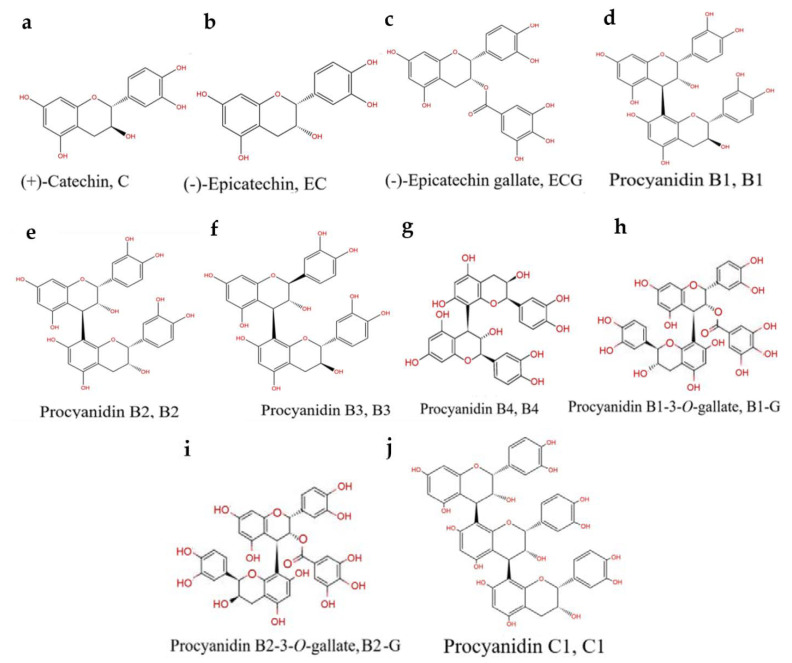
PCs structure: (**a**) (+)-Catechin (C). (**b**) (-)-Epicatechin (EC). (**c**) (-)-Epicatechin gallate (ECG). (**d**) Procyanidin B1 (B1). (**e**) Procyanidin B2 (B2). (**f**) Procyanidin B3 (B3). (**g**) Procyanidin B4 (B4). (**h**) Procyanidin B1-3-*O*-gallate (B1-G). (**i**) Procyanidin B2-3-O-gallate (B2-G). (**j**) Procyanidin C1 (C1).

Procyanidins are a natural nutrient that exhibit a strong antioxidant capacity [11]. Previous studies found that procyanidins have a protective effect in the rat pheochromocytoma cell line (PC12) and a zebrafish PD model [12]. However, the bioavailability of procyanidins is affected by their structure [13]. The number of procyanidins is large, the separation conditions are complex, and purification is difficult [14,15]. Previous studies mainly focused on mixtures of procyanidins, and there are few studies focused on the relationship between the structure of procyanidins and the prevention of PD. The structural types of procyanidins in different plants are distinct [14]. Grape seeds are one of the most abundant sources of procyanidins in nature [16]. Procyanidins in grape seeds are mainly B-type procyanidins connected by C4–C8 or C4–C6 bonds [16]. The higher the number of procyanidin structural units and the more isomers, the more difficult they are to identify and separate. Due to a lack of effective analysis methods, it is difficult to obtain pure procyanidin polymers with a high degree of polymerization [14,15]. Therefore, 10 types of pure procyanidins from grape seeds that can be obtained by current purification technology were selected to preliminarily study the relationship between the neuroprotective effect of procyanidins and their structure (Figure 1).

The nuclear factor-erythroid 2-related factor (Nrf2)/antioxidant response element (ARE) pathway plays an important protective role in PD. Activation of the Nrf2 gene was shown to alleviate the loss of dopaminergic neurons and resulted in damage to motor function [17]. NAD(P)H: quinone oxidoreductase 1 (NQO1), and heme oxygenase-1 (HO-1) are downstream of the Nrf2/ARE pathway. NQO1 is an antioxidant enzyme that uses nicotinamide adenine dinucleotide (NADH) or nicotinamide adenine dinucleotide phosphate (NADPH) as reductive cofactors and participates in the double-electron reduction of endogenous quinones [18]. Chaperone HO-1 acts synergistically with cytochrome P450 to catalyze the degradation of heme to biliverdin, which is then converted to bilirubin. Biliverdin and bilirubin both exhibit antioxidant and immunomodulatory properties [19,20]. High expression of the NQO1 and HO-1 proteins can eliminate free radicals and enhance the resistance of cells to neurotoxicity [21,22]. The PC12 cell line is highly consistent with the characteristics of primary cultured neurons and the cells are like neurons in their morphology, structure, and function [23,24]. This model has been widely used as an in vitro model in the study of nerve cells [25,26,27]. In addition, approximately 87% of zebrafish genes are homologous with human disease genes, and their neurobehavioral phenotypes are like that of human beings [28,29,30,31]. The neurotransmitter system of zebrafish, including the cholinergic, dopaminergic, and noradrenergic pathways, has been described, showing this to be a suitable animal model for the study of nervous system diseases [32,33]. Deprenyl is a safe and valuable adjunct to the conventional treatment of PD [34] and was therefore used as a positive control in this study. In this study, PC12 cells were used to establish an in vitro PD model, and zebrafish were used to establish an in vivo PD model to study whether procyanidins from grape seeds with different structures can prevent PD by regulating the Nrf2/ARE pathway.

## 2. Materials and Methods

### 2.1. Chemicals and Reagents

Catechin (C), epicatechin (EC), epicatechin gallate (ECG), procyanidin B1 (B1), procyanidin B2 (B2), procyanidin B3 (B3), procyanidin B4 (B4), procyanidin B1-3-*O*-gallate (B1-G), procyanidin B2-3-*O*-gallate (B2-G), and procyanidin C1 (C1) were obtained from Caoyuankang Biotechnology (Caoyuankang, Cheng Du, China). 1-Methyl-4-phenyl-1,2,3,6-tetrahydropyridine (MPTP) 1-methyl-4-phenylpyridinium (MPP^+^) agents were purchased from Sigma (Sigma, St. Louis, MO, USA), and CCK-8 was purchased from Beyotime (Beyotime, Shanghai, China). 2′,7′-Dichlorofluorescin diacetate, malondialdehyde (MDA), glutathione peroxidase (GSH-Px), catalase (CAT), and superoxide dismutase (SOD) diagnostic kits were purchased from Solarbio (Solarbio, Beijing, China). The cytotoxicity detection kit was purchased from Nanjing Jiancheng Institute of Bioengineering (Nanjing Jiancheng, Nanjing, China). Nrf2-siRNA, control-siRNA, and lipofectamine 2000 were obtained from Invitrogen (Invitrogen, Carlsbad, CA, USA). The following primary antibodies and corresponding secondary antibodies were obtained from Proteintech: Nrf2, HO-1, NAD(P)H: NQO1, B-cell lymphoma-2 (Bcl-2), Bax, lamin B, and GAPDH (Proteintech, Wuhan, China).

### 2.2. Cell Culture

PC12 cells were obtained from the National Collection of Authenticated Cell Cultures. The cells were maintained in DMEM medium supplemented with 10% fetal bovine serum and penicillin-streptomycin (100 U/mL; 100 μg/mL) in an incubator containing 5% CO_2_ and 95% humidity at 37 °C.

### 2.3. Cell Viability and Membrane Integrity Assays

PC12 cells were incubated with C (2.5 μM and 5 μM), EC (2.5 μM and 5 μM), ECG (2.5 μM and 5 μM), B1 (2.5 μM and 5 μM), B2 (2.5 μM and 5 μM), B3 (2.5 μM and 5 μM), B4 (2.5 μM and 5 μM), B1-G (2.5 μM and 5 μM), B2-G (2.5 μM and 5 μM), C1 (2.5 μM and 5 μM), or deprenyl (30 μM) for 24 h and then incubated with MPP^+^ (1.5 mM) for 24 h. Cell viability was analyzed via CCK-8 colorimetric assay. CCK-8 solution (10 μL) was added to each well and plates were incubated for 1 h, after which the absorbance was measured at 450 nm. Membrane integrity was analyzed by lactate dehydrogenase (LDH) release assay. LDH release was determined using a cytotoxicity detection kit, which is based on the LDH-catalyzed reduction of pyruvate to lactate by an equimolar amount of NADH. The NADH level was measured through the release of a chromogen, which emitted at 490 nm.

### 2.4. Fish Maintenance and the Experimental Group

Wild-type AB strain zebrafish were raised in an environment of 28 °C, a 10 h/14 h light/dark cycle (light/dark), and were fed once in the morning and once in the evening. The day before collecting the eggs, they were placed into a mating box at a male to female ratio of 1:1; the partition was removed on the morning of the following day, and the fish eggs were collected and cultured in an incubator at 28.5 °C. Three days after fertilization, zebrafish larvae that had developed normally were randomly divided into six groups: Control, Blank control group; Model, MPTP (400 μM); Positive control, Deprenyl (40 μM) + MPTP (400 μM); C: C (25 μM) + MPTP (400 μM); EC: EC (25 μM) + MPTP (400 μM); ECG: ECG (25 μM) + MPTP (400 μM); B1: B1 (25 μM) + MPTP (400 μM); B2: B2 (25 μM) + MPTP (400 μM); B3: B3 (25 μM) + MPTP (400 μM); B4: B4 (25 μM) + MPTP (400 μM); B1-G: B1-G (25 μM) + MPTP (400 μM); B2-G: B2-G (25 μM) + MPTP (400 μM); C1: C1 (25 μM) + MPTP (400 μM). The corresponding reagent of each treatment group was added and treated for 4 days. The ethics certificate approval no. is zju20200125.

### 2.5. ROS Measurements

PC12 cells (2 × 10^4^ cells/well) were exposed to C, EC, ECG, B1, B2, B3, B4, B1-G, B2-G, C1, or deprenyl (30 μM) for 24 h and then incubated with 1.5 mM MPP^+^ for 24 h. The cells were exposed to 10 μM 2′,7′-Dichlorofluorescin diacetate solution in dark conditions for 30 min; the dye solution was then removed, and the cells were washed with phosphate-buffered saline (PBS) three times. The images of the cells were observed using an Olympus laser scanning confocal microscope. The fluorescence intensity was quantified via ImageJ software.

### 2.6. Assessment of MDA, GSH-Px, SOD, and CAT

PC12 cells (2 × 10^4^ cells/well) were exposed to C, EC, ECG, B1, B2, B3, B4, B1-G, B2-G, C1, or deprenyl for 24 h and then incubated with 1.5 mM MPP^+^ for 24 h. Then, 1 mL of extract was added to PC12 cells, and the cells were broken by ultrasonic centrifuging at 8000 rpm at 4 °C for 10 min. The supernatant was then put on ice for testing, and the reagents were added for the determination of MDA, GSH-Px, SOD, and CAT.

The zebrafish larvae at 3 days after fertilization were incubated with deprenyl (40 μM) or C, EC, ECG, B1, B2, B3, B4, B1-G, B2-G, C1 with 400 μM MPTP for 4 days. Then, 0.05 g of zebrafish larvae tissue and 0.5 mL of extract were homogenized in an ice bath and then centrifuged at 4 °C for 10 min. Reagents were subsequently added for the determination of MDA, GSH-Px, SOD, and CAT.

### 2.7. Nrf2 siRNA Transfection

The Nrf2-siRNA sequences were as follows: forward, 5′-UUAAGACACUGUAACUCGGGAAUGG-3′; and reverse, 5′-CCAUUCCCGAGUUACAGUGUCUUA-3′. The control-siRNA sequences were as follows: 5′-UAGCGACUAAACACAUCAAUU-3′; and reverse, 5′-AAUUGAUGUGUUUAGUCGCUA-3′ (Invitrogen, Carlsbad, USA). PC12 cells were inoculated into six-well plates (2 × 10^5^ cells/well) and after the cells were adhered to the wall, the cells were washed with serum-free and antibody free Opti-MEM cell culture medium three times, and transfected with Nrf2-siRNA (80 nM) or control-siRNA using Lipofectamine 2000, according to the manufacturer’s instructions. The transfected cells were combined with C, EC, ECG, B1, B2, B3, B4, B1-G, B2-G, C1, or deprenyl for 24 h and then incubated with 1.5 mM MPP^+^ for 24 h.

### 2.8. Preparation of Whole Cell, Cytoplasmic, and Nuclear Proteins

For whole cell protein extraction, cells were collected and incubated with RIPA lysis buffer containing 1% PMSF and 1% protease inhibitor cocktail for 30 min on ice. Cell lysates were centrifuged, and the supernatant was collected and stored. For subcellular fractionation preparation, cell samples were processed using a nuclear and cytoplasmic protein extraction kit according to the manufacturer’s instructions. The protein content was assayed using BCA assay.

### 2.9. Western Blotting

Aliquoted protein samples were resolved by SDS-PAGE and transferred to polyvinylidene-difluoride membranes. The blots were then incubated with the following primary antibodies: Nrf2, HO-1, NQO1, Bcl-2, Bax, Lamin B, or GAPDH. The membranes were then washed with TBST for 5 min; this was repeated five times. Thereafter, blots were incubated in peroxidase-conjugated secondary antibodies. Finally, protein bands were visualized using an ECL-plus Western blotting detection reagent.

### 2.10. Behavioral Detection of Zebrafish

One zebrafish larva was placed in each well of a 24-well plate and allowed to adapt for 1 h. Then, the movement of each fish was recorded within 10 min and its movement track and distance were analyzed.

### 2.11. Zebrafish Tyrosine hydroxylase (TH) Immunostaining

Zebrafish larvae at 1 day after fertilization were incubated with deprenyl (40 µM) or 25 µM of procyanidins with 400 µM MPTP for 2 days (10 fish/group batches). The treated zebrafish larvae were washed with phosphate-buffered saline Tween-20 for 5 min, treated with acetone at −20 °C for 7 min, and then blocked for 1 h. After incubating the primary antibody at room temperature (dilution ratio 1:200) for 2 hours, it was rinsed with phosphate-buffered saline Tween-20 for six times for 30 min each time. The second antibody, coralite488 conjugated affinity Goat anti rabbit IgG (H + L) (dilution ratio 1:500), was incubated at room temperature in the dark for 1 h and photographed.

### 2.12. Total RNA Extraction, Reverse Transcription, and Quantitative Real-Time Polymerase Chain Reaction

RNA was extracted using RNAiso following the manufacturer’s instructions. RNA was reverse transcribed using a PrimeScript RT Kit with gDNA eraser following the manufacturer’s instructions. Quantitative real-time PCR was performed with an Applied Biosystems Vii-7 Real-Time PCR system using SYBR Premix Ex Taq II. β-actin was used as a reference gene, and relative gene expression was calculated using the 2^−∆∆CT^ method. The primer sequences utilized are listed in Table 1.

### 2.13. Statistical Analysis

Data are shown as the mean ± standard deviation (SD) of at least three independent experiments. One-way analysis of variance (ANOVA) and Duncan’s multiple range test were used to determine statistical significance, and differences were considered significant at *p* < 0.05. Statistical analyses were performed via SPSS, version 20.0.

## 3. Results

### 3.1. Effect of Different Structural Procyanidins on MPP^+^-Induced PC12 Cell Damage

**Figure 2:**
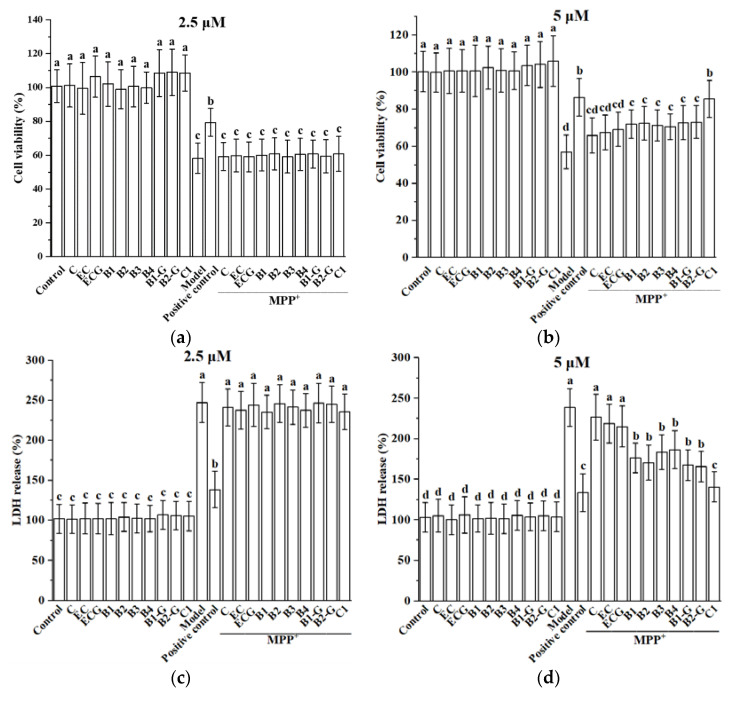
Effect of different structural procyanidins on MPP^+^-induced PC12 cell damage. (**a**) Effect of 2.5 μM procyanidins with different structures on the survival of MPP^+^-injured PC12 cells. (**b**) Effect of 5 μM procyanidins with different structures on the survival of MPP^+^-injured PC12 cells. (**c**) Effect of 2.5 μM procyanidins with different structures on MPP^+^-induced LDH release. (**d**) Effect of 5 μM procyanidins with different structures on MPP^+^-induced LDH release. Control, Blank control group; Model, MPP^+^ (1.5 mM); Positive control, Deprenyl (30 μM) + MPP^+^ (1.5 mM). Data are expressed as the mean ± SD. All experiments were conducted six times. Different letters (a–d) on the bar represent significant differences, while the same letters represent no significant differences (*p* < 0.05, one-way ANOVA).

PC12 cells were injured via treatment with 1.5 mM MPP^+^ for 24 h, resulting in decreased cell viability. Pre-treatment with 2.5 μM and 5 μM procyanidin monomers C, EC, ECG, procyanidin dimers B1, B2, B3, B4, B1-G, B2-G, and the procyanidin trimer C1 had no effect on cell viability. However, pre-treatment with 5 μM procyanidin dimers and the procyanidin trimer C1 treatment significantly improved cell viability (Figure 2a,b, *p* < 0.05 compared with the model group). However, treatment with 2.5 μM procyanidin monomers, procyanidin dimers, and the procyanidin trimer C1 had no significant protective effect on MPP^+^-injured PC12 cells. LDH is a soluble cytosolic enzyme that is released into the culture medium following loss of membrane integrity resulting from cell damage. To confirm the cytoprotection of different structural procyanidins, we further determined the content of LDH leakage after MPP^+^ insult. The experimental results showed that 5 μM procyanidin dimers and the procyanidin trimer C1 treatment significantly reduced the leakage of LDH, while 2.5 μM procyanidin monomers, procyanidin dimers, and the procyanidin trimer C1 treatment had no significant effect on preventing LDH leakage (Figure 2c,d, *p* < 0.05 compared with the model group). Therefore, 5 μM of procyanidin monomers, dimers, and the trimer C1 were selected for further study.

### 3.2. Effects of Different Structural Procyanidins on the Expression of Bax and Bcl-2 Proteins in the PC12 Cell PD Model

**Figure 3:**
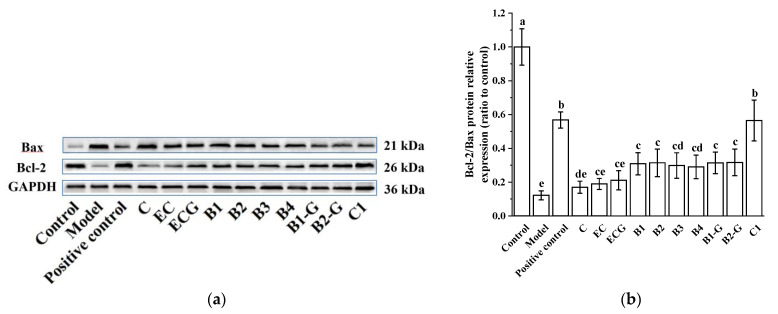
Effects of different structural procyanidins on the expression of Bax and Bcl-2 proteins in the PC12 cell PD model. (**a**) Protein levels of Bax and Bcl-2, as determined by Western blotting. (**b**) Bcl-2/Bax protein relative expression (ratio to control). Control, Blank control group; Model, MPP^+^ (1.5 mM); Positive control, Deprenyl (30 μM) + MPP^+^ (1.5 mM); C: C (5 μM) + MPP^+^ (1.5 mM); EC: EC (5 μM) + MPP^+^ (1.5 mM); ECG: ECG (5 μM) + MPP^+^ (1.5 mM); B1: B1 (5 μM) + MPP^+^ (1.5 mM); B2: B2 (5 μM) + MPP^+^ (1.5 mM); B3: B3 (5 μM) + MPP^+^ (1.5 mM); B4: B4 (5 μM) + MPP^+^ (1.5 mM); B1-G: B1-G (5 μM) + MPP^+^ (1.5 mM); B2-G: B2-G (5 μM) + MPP^+^ (1.5 mM); C1: C1(5 μM) + MPP^+^ (1.5 mM). Data are expressed as the mean ± SD. All experiments were conducted six times. Different letters (a–e) on the bar represent significant differences, while the same letters represent no significant differences (*p* < 0.05, one-way ANOVA).

Bcl-2 and Bax proteins are key proteins in the Bcl-2 family and play different roles in the process of apoptosis. Bcl-2 is an anti-apoptotic protein that promotes cell survival, while Bax promotes apoptosis [35]. As shown in Figure 3a,b, MPP^+^-injured PC12 cells exhibited increased expression of Bax and decreased expression of Bcl-2. Procyanidin dimer and procyanidin trimer C1 treatment significantly downregulated Bax protein expression and upregulated Bcl-2 protein expression and had a significant protective effect on MPP^+^-injured PC12 cells. There was no significant difference between the procyanidin dimer treatment groups.

### 3.3. Effects of Different Structural Procyanidins on Oxidative Stress in the PC12 Cell PD Model

**Figure 4:**
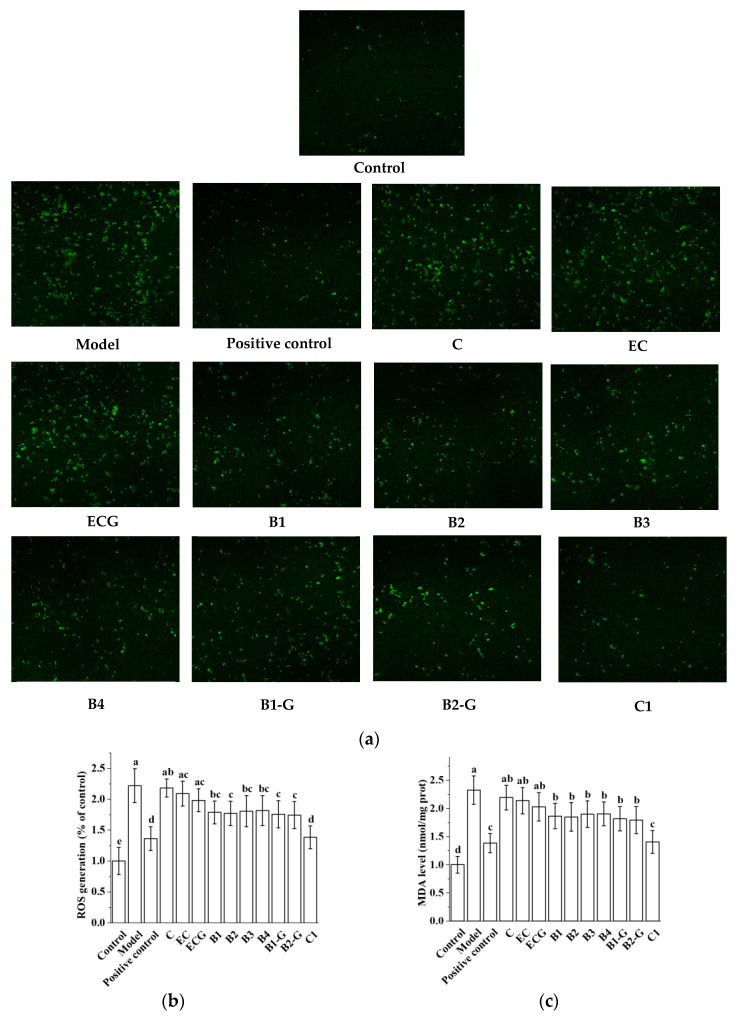

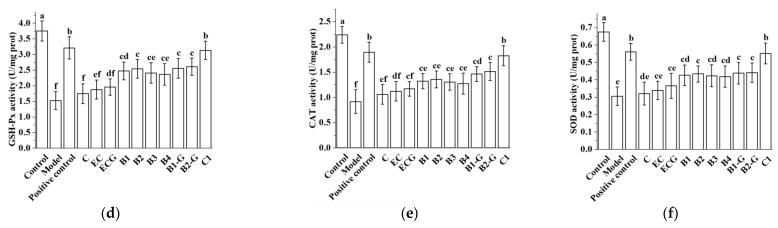
Effects of different structural procyanidins on oxidative stress in the PC12 cell PD model. (**a**) Representative fluorescence photomicrographs of PC12 cells. (**b**) ROS level. (**c**) MDA level. (**d**,**e**) CAT activity. (**f**) SOD activity. Control, Blank control group; Model, MPP^+^ (1.5 mM); Positive control, Deprenyl (30 μM) + MPP^+^ (1.5 mM); C: C (5 μM) + MPP^+^ (1.5 mM); EC: EC (5 μM) + MPP^+^ (1.5 mM); ECG: ECG (5 μM) + MPP^+^ (1.5 mM); B1: B1 (5 μM) + MPP^+^ (1.5 mM); B2: B2 (5 μM) + MPP^+^ (1.5 mM); B3: B3 (5 μM) + MPP^+^ (1.5 mM); B4: B4 (5 μM) + MPP^+^ (1.5 mM); B1-G: B1-G (5 μM) + MPP^+^ (1.5 mM); B2-G: B2-G (5 μM) + MPP^+^ (1.5 mM); C1: C1(5 μM) + MPP^+^ (1.5 mM). Data are expressed as the mean ± SD. All experiments were conducted three times. Different letters (a–f) on the bar represent significant differences, while the same letters represent no significant differences (*p* < 0.05, one-way ANOVA).

The content of ROS and MDA is an important index for evaluating oxidative stress [36,37,38]. After PC12 cells were injured by 1.5 mM MPP^+^ for 24 h, the ROS and MDA content in PC12 cells was increased. Pre-treatment with procyanidin dimers and the procyanidin trimer C1 inhibited the increase in ROS and MDA induced by MPP^+^ injury (Figure 4a–c, *p* < 0.05 versus the model group). There was no significant difference in the content of ROS and MDA between the procyanidin monomer treatment groups and the model group (Figure 4a–c, *p* > 0.05), and there was no significant difference between the procyanidin trimer C1 treatment group and the positive control group (Figure 4a–c, *p* > 0.05). There was no significant difference between the C, EC, and ECG treatment groups, and there was also no significant difference between the B1, B2, B3, B4, B1-G, and B2-G treatment groups (Figure 4a–c, *p* > 0.05). GSH-Px, CAT, and SOD are important components of the antioxidant system, which can antagonize and block free radicals [39,40,41]. Detection of the activities of GSH-Px, CAT, and SOD can reflect the antioxidant capacity of cells. The results showed that MPP^+^ treatment inhibited the activities of GSH-Px, CAT, and SOD. However, MPP^+^-induced effects were decreased by pre-treatment with procyanidin dimers and the procyanidin trimer C1 (Figure 4d–f, *p* < 0.05 versus the model group). There was no significant difference between the procyanidin dimer treatment groups (Figure 4d–f, *p* > 0.05).

### 3.4. Effects of Different Structural Procyanidins on the Nrf2/ARE Pathway in the PC12 Cell PD Model

**Figure 5:**
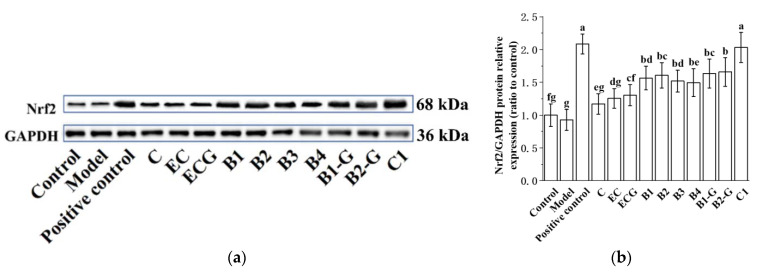

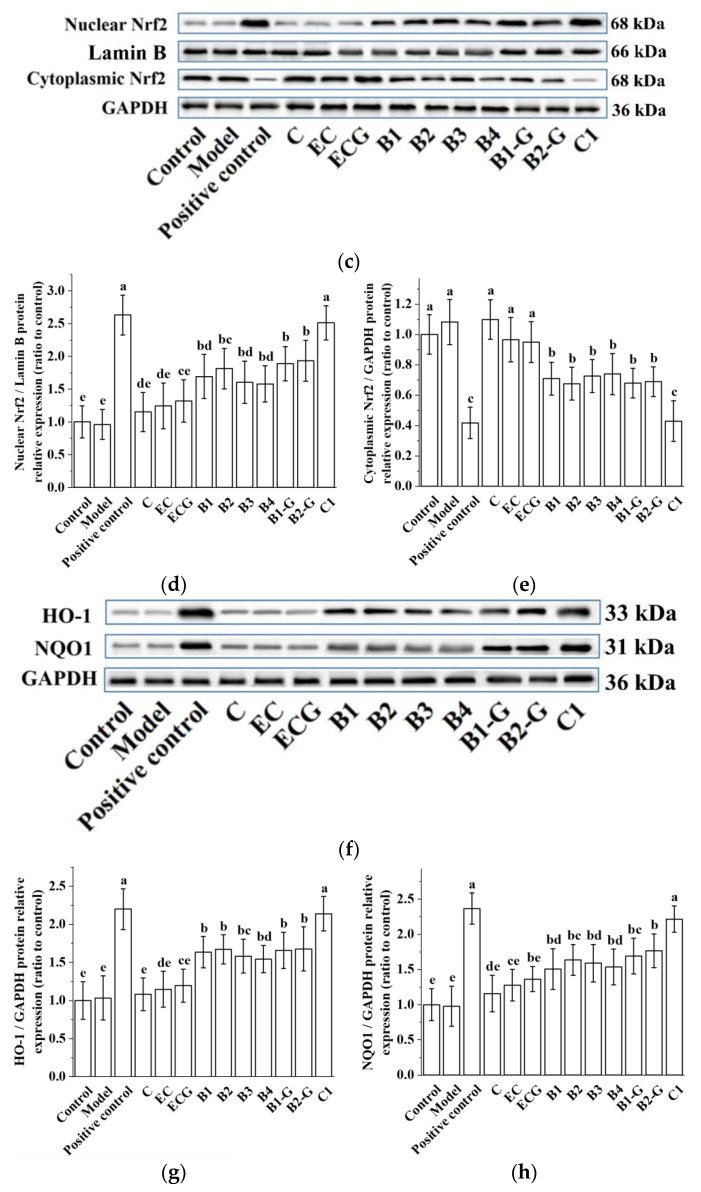
Effects of different structural procyanidins on the nuclear factor-erythroid 2-related factor 2 (Nrf2)/ARE Pathway in the PC12 cell PD model. (**a**) Protein levels of Nrf2, as determined by Western blotting. (**b**) Nrf2/GAPDH protein relative expression (ratio to control). (**c**) Protein expression levels of nuclear Nrf2 and cytoplasmic Nrf2, as determined by Western blotting. (**d**) Nuclear Nrf2/LaminB protein relative expression (ratio to control). (**e**) Cytoplasmic Nrf2/GAPDH protein relative expression (ratio to control). (**f**) Protein levels of HO-1 and NQO1, as determined by Western blotting; (**g**) HO-1/GAPDH protein relative expression (ratio to control). (**h**) NQO1/GAPDH protein relative expression (ratio to control). Control, Blank control group; Model, MPP^+^ (1.5 mM); Positive control, Deprenyl (30 μM) + MPP^+^ (1.5 mM); C: C (5 μM) + MPP^+^ (1.5 mM); EC: EC (5 μM) + MPP^+^ (1.5 mM); ECG: ECG (5 μM) + MPP^+^ (1.5 mM); B1: B1 (5 μM) + MPP^+^ (1.5 mM); B2: B2 (5 μM) + MPP^+^ (1.5 mM); B3: B3 (5 μM) + MPP^+^ (1.5 mM); B4: B4 (5 μM) + MPP^+^ (1.5 mM); B1-G: B1-G (5 μM) + MPP^+^ (1.5 mM); B2-G: B2-G (5 μM) + MPP^+^ (1.5 mM); C1: C1(5 μM) + MPP^+^ (1.5 mM). Data are expressed as the mean ± SD. All experiments were conducted three times. Different letters (a–g) on the bar represent significant differences, while the same letters represent no significant differences (*p* < 0.05, one-way ANOVA).

The Nrf2/ARE pathway is an important antioxidant pathway [42]. Under normal conditions, Nrf2 binds to the inhibitory protein Keap1 in the cytoplasm [43]. When subjected to oxidative damage, Keap1 is separated from Nrf2. After Nrf2 is activated, it enters the nucleus from the cytoplasm, induces the transcription of ARE-dependent antioxidant genes, and enhances antioxidant capacity [44,45,46,47,48,49]. The results showed that pre-treatment with procyanidin dimers and the procyanidin trimer C1 upregulated expression of the Nrf2 protein and increased the nuclear accumulation of Nrf2 in PC12 cells damaged by MPP^+^ (Figure 5a–e, *p* < 0.05 versus the model group). High expression of the NQO1 and HO-1 proteins can eliminate free radicals and enhance the resistance of cells to neurotoxicity [21,22]. Further evaluation of the expression of the HO-1 and NQO1 proteins showed that their expression levels were upregulated by procyanidin dimers and the procyanidin trimer C1 (Figure 5f–h, *p* < 0.05 versus the model group). There was no significant difference between the procyanidin monomer treatment groups. There was also no significant difference between the procyanidin dimer treatment groups (Figure 5a–h, *p* > 0.05).

### 3.5. Verification of the Effect of Nrf2 on the Protective Effect of Different Structural Procyanidins in the PC12 Cell PD Model

**Figure 6:**
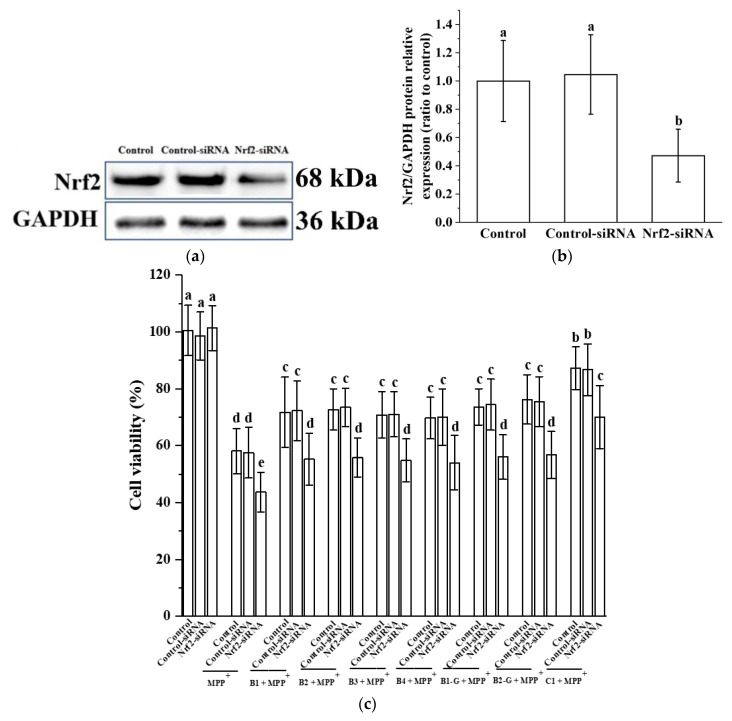
Verification of the effect of Nrf2 on the protective effect of different structural procyanidins in the PC12 cell PD model. (**a**) Knockout efficiency was detected by determination of Nrf2 protein expression using Western blotting. (**b**) Nrf2/GAPDH protein relative expression (ratio to control). (**c**) Cell viability. Data are shown as the mean ± SD. All experiments were conducted three times. Different letters (a–e) on the bar represent significant differences, while the same letters represent no significant differences (*p* < 0.05, one-way ANOVA).

In order to verify the effect of Nrf2 on the protective effect of different structural procyanidins in the PC12 cell PD model, we used siRNA to knock down the expression of Nrf2 in the PC12 cells. Knockdown was confirmed by Western blot analysis (Figure 6a,b). Nrf2 siRNA treatment inhibited the expression of the Nrf2 protein in MPP^+^-injured PC12 cells and eliminated the protective effects of procyanidin dimers and the procyanidin trimer C1, as evidenced by decreased cell viability in the Nrf2 siRNA transfection group (Figure 6c).

### 3.6. Effects of Different Structural Procyanidins on Exercise Capacity in a Zebrafish PD Model

**Figure 7:**
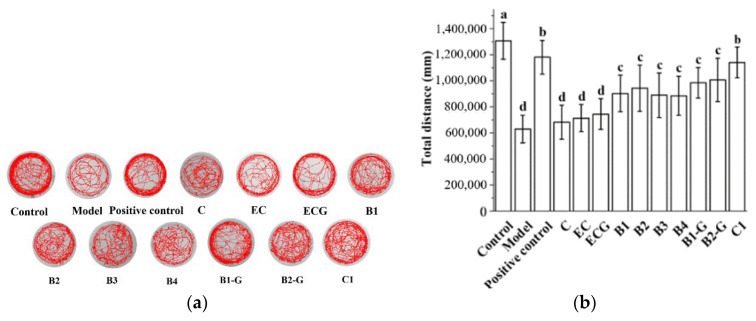
Effects of different structural procyanidins on exercise capacity in the zebrafish PD model. (**a**) Swimming traces of zebrafish in each group. (**b**) Average total distance of zebrafish in each group. Control, Blank control group; Model, MPTP (400 μM); Positive control, Deprenyl (40 μM) + MPTP (400 μM); C: C (25 μM) + MPTP (400 μM); EC: EC (25 μM) + MPTP (400 μM); ECG: ECG (25 μM) + MPTP (400 μM); B1: B1 (25 μM) + MPTP (400 μM); B2: B2 (25 μM) + MPTP (400 μM); B3: B3 (25 μM) + MPTP (400 μM); B4: B4 (25 μM) + MPTP (400 μM); B1-G: B1-G (25 μM) + MPTP (400 μM); B2-G: B2-G (25 μM) + MPTP (400 μM); C1: C1 (25 μM) + MPTP (400 μM). Data are expressed as the mean ± SD. All experiments were conducted three times. Different letters (a–d) on the bar represent significant differences, while the same letters represent no significant differences (*p* < 0.05, one-way ANOVA).

Previous research in our laboratory found that 25 μM of 10 grape seed-derived procyanidins did not decrease the exercise ability of zebrafish and improved the movement behavior disorder caused by H_2_O_2_ [50]. Therefore, this study continued to use 25 μM of 10 grape seed-derived procyanidins to study the effects of different structural procyanidins on the exercise capacity of the zebrafish PD model. This study found that 25 μM grape seed derived procyanidins had a protective effect on the exercise ability of zebrafish damaged by MPTP. The protective effects observed in the procyanidin dimer treatment groups and procyanidin trimer C1 treatment group were significantly different from the model group (Figure 7a,b, *p* < 0.05 versus the model group). There was no significant difference between the procyanidin haploid treatment groups with different structures. There was no significant difference between the procyanidin dimer treatment groups with different structures (Figure 7a,b, *p* > 0.05).

### 3.7. Effects of Different Structural Procyanidins on Dopaminergic Neuron Injury in the Zebrafish PD Model

**Figure 8:**
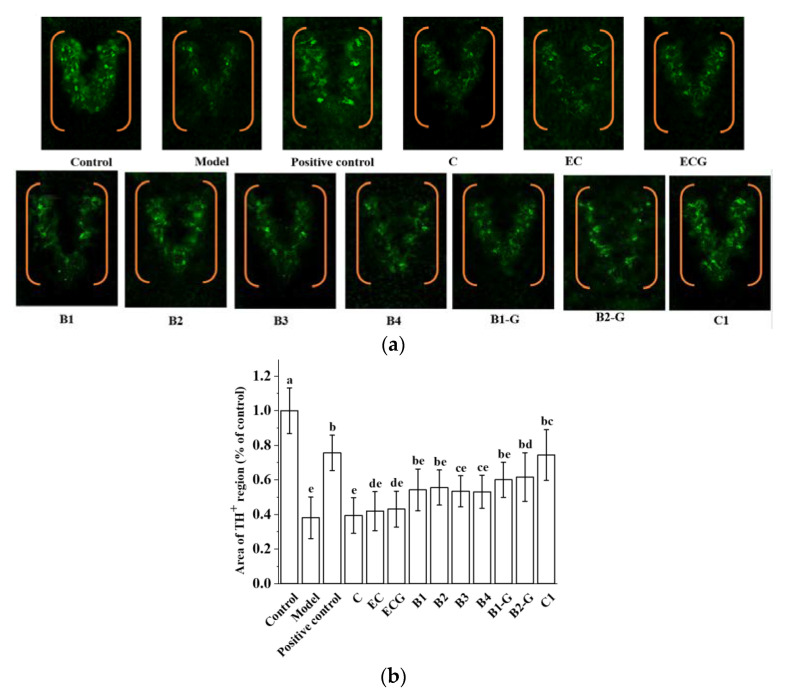
Effects of different structural procyanidins on dopaminergic neuron injury in the zebrafish PD model. (**a**) Representative pictures of the dopaminergic neurons in the brains of zebrafish. (**b**) Quantitative analysis of the tyrosine hydroxylase (TH) density. Control, Blank control group; Model, MPTP (400 μM); Positive control, Deprenyl (40 μM) + MPTP (400 μM); C: C (25 μM) + MPTP (400 μM); EC: EC (25 μM) + MPTP (400 μM); ECG: ECG (25 μM) + MPTP (400 μM); B1: B1 (25 μM) + MPTP (400 μM); B2: B2 (25 μM) + MPTP (400 μM); B3: B3 (25 μM) + MPTP (400 μM); B4: B4 (25 μM) + MPTP (400 μM); B1-G: B1-G (25 μM) + MPTP (400 μM); B2-G: B2-G (25 μM) + MPTP (400 μM); C1: C1 (25 μM) + MPTP (400 μM). Data are expressed as the mean ± SD. All experiments were conducted three times. Different letters (a–e) on the bar represent significant differences, while the same letters represent no significant differences (*p* < 0.05, one-way ANOVA).

Common pathological changes in the brains of PD patients include the degeneration and death of dopaminergic neurons in the substantia nigra and a low level of dopamine neurotransmitter in the substantia nigra striatum. TH is the key enzyme of the dopamine biosynthesis pathway. The detection of TH activity can indirectly reflect the apoptosis of dopaminergic neurons. This is an effective index for evaluating the occurrence and development of PD [51]. The density of TH in the zebrafish brain in the B2-G and C1 treatment groups was higher than that in the model group (Figure 8a,b, *p* < 0.05). This shows that B2-G and C1 can reduce the apoptosis of dopaminergic neurons in the zebrafish brain to a certain extent, and the effect on the B2-G and C1 treatment groups was greater than that in the other treatment groups.

### 3.8. Effects of Different Structural Procyanidins on Oxidative Stress in the Zebrafish PD Model

**Figure 9:**
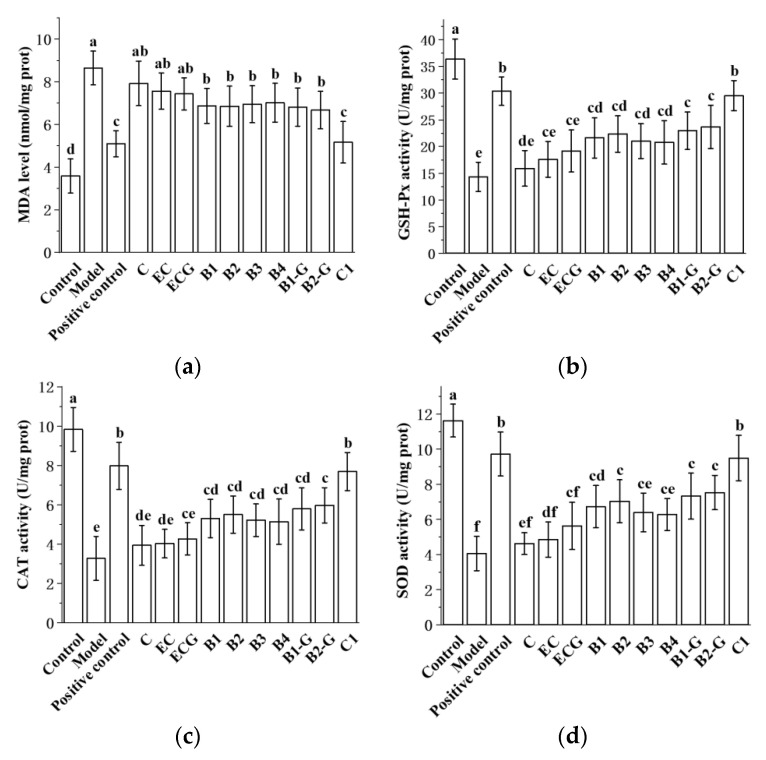
Effects of different structural procyanidins on oxidative stress in the zebrafish PD model. (**a**) MDA levels. (**b**) GSH-Px activity. (**c**) CAT activity. (**d**) SOD activity. Control, Blank control group; Model, MPTP (400 μM); Positive control, Deprenyl (40 μM) + MPTP (400 μM); C: C (25 μM) + MPTP (400 μM); EC: EC (25 μM) + MPTP (400 μM); ECG: ECG (25 μM) + MPTP (400 μM); B1: B1 (25 μM) + MPTP (400 μM); B2: B2 (25 μM) + MPTP (400 μM); B3: B3 (25 μM) + MPTP (400 μM); B4: B4 (25 μM) + MPTP (400 μM); B1-G: B1-G (25 μM) + MPTP (400 μM); B2-G: B2-G (25 μM) + MPTP (400 μM); C1: C1 (25 μM) + MPTP (400 μM). Data are expressed as the mean ± SD. All experiments were conducted three times. Different letters (a–f) on the bar represent significant differences, while the same letters represent no significant differences (*p* < 0.05, one-way ANOVA).

The content of MDA in different procyanidin monomer treatment groups was lower than in the model group, and the activities of antioxidant enzymes (GSH-Px, CAT, SOD) were higher than in the model group. There was no significant difference between the procyanidin monomer treatment groups and the model group (Figure 9a–d, *p* > 0.05), but there was a significant difference between the procyanidin dimer treatment groups, the procyanidin trimer C1 treatment group, and the model group (Figure 9a–d, *p* < 0.05). The effect of the procyanidin trimer C1 treatment in reducing MDA content and increasing the activity of antioxidant enzymes (GSH-Px, CAT, SOD) was higher than in the other treatment groups (Figure 9a–d). There was no significant difference between the procyanidin monomer treatment groups. There was also no significant difference between the procyanidin dimer treatment groups (Figure 9a–d, *p* > 0.05).

### 3.9. Effects of Different Structural Procyanidins on the Nrf2/ARE Pathway in the Zebrafish PD Model

**Figure 10:**
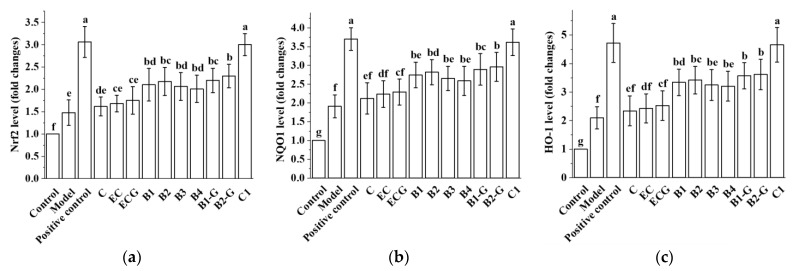
Effects of different structural procyanidins on the Nrf2/ARE pathway in the zebrafish PD model. (**a**) Nrf2 levels. (**b**) NQO1 levels. (**c**) HO-1 levels. Control, Blank control group; Model, MPTP (400 μM); Positive control, Deprenyl (40 μM) + MPTP (400 μM); C: C (25 μM) + MPTP (400 μM); EC: EC (25 μM) + MPTP (400 μM); ECG: ECG (25 μM) + MPTP (400 μM); B1: B1 (25 μM) + MPTP (400 μM); B2: B2 (25 μM) + MPTP (400 μM); B3: B3 (25 μM) + MPTP (400 μM); B4: B4 (25 μM) + MPTP (400 μM); B1-G: B1-G (25 μM) + MPTP (400 μM); B2-G: B2-G (25 μM) + MPTP (400 μM); C1: C1 (25 μM) + MPTP (400 μM). Data are expressed as the mean ± SD. All experiments were conducted three times. Different letters (a–g) on the bar represent significant differences, while the same letters represent no significant differences (*p* < 0.05, one-way ANOVA).

Different structural procyanidins upregulated the expression of Nrf2, NQO1, and HO-1 genes in the zebrafish model induced by MPTP. There was no significant difference between the procyanidin monomer treatment groups and the model group (Figure 10a–c, *p* > 0.05). No significant difference was observed between the procyanidin monomer C, EC, and ECG treatment groups (Figure 10a–c, *p* > 0.05). However, there was a significant difference between the procyanidin dimer treatment groups, the procyanidin trimer C1 treatment group, and the model group (Figure 10a–c, *p* < 0.05). No significant difference was observed between the procyanidin dimer B1, B2, B3, B4, B1-G, and B2-G treatment groups (Figure 10a–c, *p* > 0.05). Upregulation of the Nrf2, NQO1, and HO-1 genes in the procyanidin trimer C1 treatment group was greater than in the other treatment groups (Figure 10a–c). This was consistent with the research results in the PC12 cell model.

## 4. Discussion

PD is a neurodegenerative disease caused by a loss of dopaminergic neurons in the substantia nigra [2]. The pathogenesis of PD is not yet clear; however, oxidative stress may be a primary PD cause [52,53]. Therefore, supplementation with antioxidants may be a feasible strategy for preventing PD. Procyanidins are natural nutrients and antioxidants. In the female Swiss-Webster mouse model (induced by 12-*O*-tetradecanoylphorbol-13-acetate), the antioxidant capacity of procyanidins was stronger than that of vitamin C, vitamin E succinate, and β-carotene [54]. Studies found that a mixture of procyanidins exhibited a protective effect in a zebrafish PD model [12]. However, the relationship between the preventive effect of procyanidins and their structural characteristics is not clear. PC12 cells are similar to neurons in morphology, structure, and function [23,24], and are widely used as an in vitro model in neural cell research [25,26,27]. The neurotransmitter system of zebrafish, including the cholinergic, dopaminergic, and noradrenergic pathways, has been clarified and is a suitable animal model for the study of nervous system diseases [32,33]. Therefore, we utilized PC12 cells as an in vitro PD model and zebrafish as an in vivo PD model to study the relationship between the structures of grape seed-derived procyanidins and the prevention of PD.

When we treated PC12 cells first with MPP^+^ and then with procyanidins in the pre-experiment, the experimental results showed that procyanidins were not protective. Therefore, we chose to treat PC12 cells with procyanidins first and then with MPP^+^ in this study. The results showed that, compared with the model group, 5 μM procyanidin dimers and the procyanidin trimer C1 had a protective effect on PC12 cells damaged by MPP^+^ (*p* < 0.05). However, there was no significant difference between the 5 μM procyanidin monomer treatment groups and the model group (*p* > 0.05). Procyanidin dimers and the procyanidin trimer C1 decreased the damage to dopaminergic neurons and motor impairment in the zebrafish PD model. The effect of the procyanidin trimer C1 was better than that of other treatment groups. These results show that the position of -OH and -galloyl moiety did not play a role in protecting cell viability in the PC12 cell PD model or motor capacity in the zebrafish PD model. The degree of polymerization of procyanidins was positively correlated with the viability of MPP^+^-damaged PC12 cells and the exercise capacity of zebrafish in the PD model.

We further evaluated the effects of different procyanidins from grape seeds on the content of ROS and MDA, and antioxidant enzyme (GSH-Px, CAT and SOD) activity in PC12 cells and the zebrafish PD model. The results showed that, compared with the model group, procyanidin dimers and the procyanidin trimer C1 significantly reduced the ROS and MDA content and increased the activity of antioxidant enzymes (GSH-Px, CAT and SOD) (*p* < 0.05). The procyanidin monomers exerted a positive effect on the decrease in ROS and MDA and the increase in antioxidant enzyme activity in the PD model; however, the difference from the model group was not significant (*p* > 0.05). These results showed that antioxidant capacity increased with increasing polymerization of the procyanidins. The antioxidant capacity of trimer C1 was better than that of the other treatment groups, and this may have been due to the fact that trimer C1 has more structural units than dimer and monomers. There was no significant difference in antioxidant capacity between procyanidin monomer treatment groups, and there was also no significant difference in antioxidant capacity between procyanidin dimer treatment groups. This may be because the position of the -OH and -galloyl moieties had no effect on the antioxidant capacity of these procyanidins.

The Nrf2/ARE pathway plays an important protective role in PD. Activation of the Nrf2 gene alleviated the loss of dopaminergic neurons and the resulting damage to motor function [17]. In the Drosophila PD model, overexpression of Nrf2 or knockout of Keap1 delayed the loss of dopaminergic neurons [55]. In addition, the loss of striatal dopamine transporter was greater in PD model mice after knockout of the Nrf2 gene, compared with wild-type mice [56,57,58,59,60,61]. Activation of Nrf2 in astrocytes has been shown to reduce the neurotoxicity of MPP^+^ [62]. The Nrf2/ARE pathway not only has an antioxidant effect but can also regulate redox homeostasis and promote cell survival in a PD model. Therefore, this research further studied regulation of the Nrf2/ARE pathway by procyanidins with different structures in PC12 cells and the zebrafish PD model. The results showed that procyanidin dimers and the procyanidin trimer C1 upregulated expression of the Nrf2 protein, promoted the accumulation of Nrf2 in the nucleus, and upregulated expression of the phase II detoxification enzymes NQO1 and HO-1, which are downstream of the Nrf2/ARE pathway, in the PC12 cell model of PD. NQO1 is an antioxidant enzyme that uses NADH or NADPH as reductive cofactors and participates in the double-electron reduction of endogenous quinones [18]. Chaperone HO-1 acts synergistically with cytochrome P450 to catalyze the degradation of heme to biliverdin, which is then converted to bilirubin. Biliverdin and bilirubin both exhibit antioxidant and immunomodulatory properties [19,20]. High expression of the NQO1 and HO-1 proteins can eliminate free radicals and enhance the resistance of cells to neurotoxicity [21,22]. The procyanidin trimer C1 upregulated the Nrf2/ARE pathway to a greater extent than the procyanidin dimers. Nrf2 siRNA was utilized to further evaluate the role of the Nrf2/ARE pathway in the effects of the procyanidin dimers and the procyanidin trimer C1 in the PD model. The results showed that expression of the Nrf2 protein and survival of PC12 cells transfected with the Nrf2 siRNA decreased. In the zebrafish PD model, procyanidin treatment upregulated expression of the Nrf2, NQO1, and HO-1 genes. The upregulation of the Nrf2, NQO1, and HO-1 genes in the procyanidin trimer C1 treatment group was greater than in the other treatment groups. These findings suggest that the regulatory effect of procyanidins on the Nrf2/ARE pathway in the PD model was related to the degree of procyanidin polymerization and is independent from the position of the -OH and -galloyl moiety.

There were some limitations in the present study. The current in vivo experiment adopted the zebrafish juvenile model, which does not account for the bioavailability of procyanidins in mammals. Therefore, future studies will utilize mouse models and human experiments to further study the bioavailability of procyanidins. This research studied the role of the Nrf2/ARE pathway in the protective effect of procyanidins on Parkinson’s disease. In future studies, we will investigate other pathways that may be involved in the protective effect of procyanidins in Parkinson’s disease. Additionally, because the data in this study required paired comparisons, the Duncan’s multiple range test was used. However, this test is likely to introduce a type I error; therefore, we will use other data analysis methods in future research.

## 5. Conclusions

**Figure 11:**
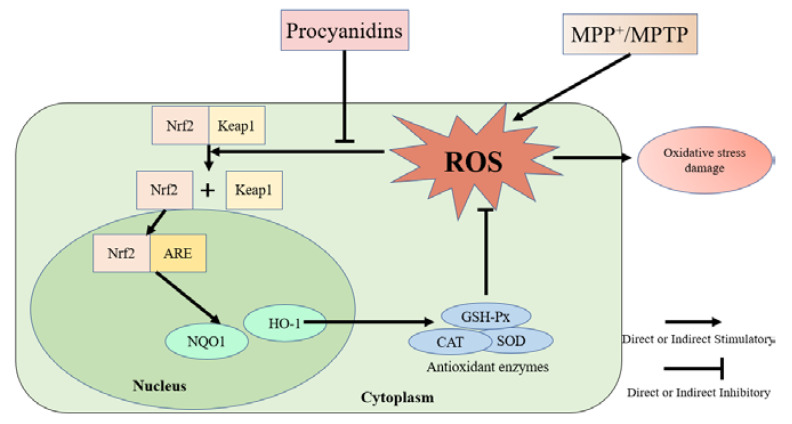
Schematic diagram of the protective effects of procyanidins in PD models.

In conclusion, procyanidins (monomers, dimers, and one trimer) from grape seeds with different structures may be useful in the prevention of PD by activating the Nrf2/ARE pathway and its downstream detoxification (NQO1, HO-1) and antioxidant enzymes (GSH-Px, CAT, and SOD) (Figure 11). The effect of the procyanidin trimer C1 treatment group was greater than that of the other treatment groups, showing that the degree of polymerization of procyanidins is an important factor affecting their neuroprotective effect. The antioxidant capacity of trimer C1 was better than that of the other treatment groups, which may have been due to the fact that trimer C1 has more structural units than dimer and monomers. There was no significant difference between the procyanidins C, EC, ECG, and B1, B2, B3, B4, B1-G, and B2-G treatment groups, indicating that the position of the -OH and -galloyl moieties in the 10 procyanidins in this study had no effect on the prevention of PD.

## Figures and Tables

**Table 1:** Sequences of primers for quantitative real-time PCR.

Genes	Forward Primer	Reverse Primer
β-Actin	CACTGAGGCTCCCCTGAATC	GGGTCACACCATCACCAGAG
Nrf2	CTGCTGTCACTCCCAGAGTT	GCCGTAGTTTTGGGTTGGTG
HO-1	AAGAGCTGGACAGAAACGCA	AGAAGTGCTCCAAGTCCTGC
NQO1	AAGCCTCTGTCCTTTGCTCC	TGCTGTGGTAATGCCGTAGG

## Data Availability

Data is contained within the article.
